# Simple Binding and Dissociation of a Sialoglycoprotein Using Boronic Acid-Modified Functional Interfaces on Microparticles

**DOI:** 10.3390/s24041080

**Published:** 2024-02-07

**Authors:** Yukichi Horiguchi, Masato Yasuura, Hiroki Ashiba, Zheng Lin Tan, Takashi Fukuda

**Affiliations:** Sensing System Research Center (SSRC), Department of Electronics and Manufacturing, National Institute of Advanced Industrial Science and Technology (AIST), Central 5, 1-1-1 Higashi, Tsukuba 305-8565, Ibaraki, Japan; yasuura-masato@aist.go.jp (M.Y.); h.ashiba@aist.go.jp (H.A.); william.tanzl94@gmail.com (Z.L.T.); t-fukuda@aist.go.jp (T.F.)

**Keywords:** cancer, sialic acid, boronic acid, magnetic particle, diagnosis

## Abstract

An overexpression of sialic acid is an indicator of metastatic cancer, and selective detection of sialic acid shows potential for cancer diagnosis. Boronic acid is a promising candidate for this purpose because of its ability to specifically bind to sialic acid under acidic conditions. Notably, the binding strength can be easily modulated by adjusting the pH, which allows for a simple dissociation of the bound sialic acid. In this study, we developed 5-boronopicolinic acid (5-BPA)-modified magnetic particles (BMPs) to selectively capture sialic acid biomolecules. We successfully captured fetuin, a well-known sialoglycoprotein, on BMPs at >10^4^ molecules/particle using an acetate buffer (pH 5.0). Facile dissociation then occurred when the system was changed to a pH 7.6 phosphate buffer. This capture-and-release process could be repeated at least five times. Moreover, this system could enrich fetuin by more than 20 times. In summary, BMPs are functional particles for facile purification and concentration through the selective capture of sialic acid proteins and can improve detection sensitivity compared with conventional methods. This technology shows potential for the detection of sialic acid overexpression by biological particles.

## 1. Introduction

Sialic acid is a critical indicator of physiological function that predominantly exists in vivo as conjugates, such as glycoproteins and gangliosides. It plays a pivotal role in the development of brain function and immunity [1,2]. An overexpression of sialic acid is an indicator of serious disease. Studies have shown a correlation between sialylation rates and metastatic potential [3], and there is evidence that inhibiting sialyltransferase can suppress metastasis [4]. Cancer metastasis involves cancer cells from a lesion evading the immune system and entering the bloodstream [5]. Matrix metalloproteinases, which are crucial enzymes in this process, are upregulated by sialyltransferase, and this increases the sialylation level of terminal glycans on glycoproteins embedded in cell membranes [6]. Therefore, a technique to measure the degree of sialylation would be valuable for assessing the potential malignancy of cancer.

Techniques for detecting the overexpression of sialic acid through extracellular vesicles have become important tools for cancer diagnosis. Exosomes, which are lipid-derived small extracellular vesicles with diameters ranging from 30 to 150 nm, participate in cell signaling through messenger RNA and micro-RNA (miRNA) [7]. This mechanism contributes to the mutation of cancer cells with miRNA in exosomes promoting processes such as the epithelial–mesenchymal transition, which is crucial for cells acquiring metastatic potential—for example, properties that facilitate invasion [8]. Moreover, miRNA in exosomes has been implicated in cancer transformation, where it influences factors such as cancer growth, the acquisition of chemoresistance capacity, and control of metastatic processes [9,10,11,12]. Consequently, exosomes are important targets in cancer research. Notably, because extracellular vesicles such as exosomes retain the original cell surface characteristics, when they are derived from cancer cells overexpressing sialic acid, they also have high sialic acid concentrations.

Conventional methods use antibodies such as CD44 and CD63 to isolate cancer cells or cancer-related substances, such as exosomes, from biological fluids (e.g., blood) for subsequent analysis [13,14,15,16]. Although this approach is highly specific, its effectiveness is contingent on the expression of the relevant antigen. The heterogeneous nature of cancer tissue, which acquires diverse characteristics during cancer growth, is challenging because certain targets may be overlooked if the associated antigen is not expressed [17]. The diversity of cancers extends to their exosomes, which can result in false positive or negative results. Additionally, antibodies are prone to gradual denaturation through hydrophobic interactions after immobilization on sensing or capturing surfaces [18]. Thus, maintaining the stability of antibodies for extended periods is challenging and leads to potential variations in sensing or capturing results.

As discussed earlier, the heterogeneity of cancer tissue complicates cancer diagnosis. Sialic acid is a promising target that is common to multiple cancer types and could be used to address the tissue complexity issue. Metastatic cancer tissues generally overexpress sialic acid. This overexpression is not limited to cancer cells but extends to proteins, exosomes, and other biomolecules released from the cells. Sialic acid collected efficiently from biological fluids could be used to assess the risk of metastatic cancer. Studies have shown that measuring sialic acid concentrations can enhance diagnostic accuracy. For instance, prostate-specific antigen (PSA), a common biomarker for prostate cancer diagnosis [19,20], is not exclusively indicative of prostate cancer because elevated PSA levels are also observed in conditions such as benign prostatic hyperplasia and prostatitis. Notably, analysis of the glycan structure on PSA, particularly the amount of sialic acid, reportedly greatly improves the accuracy of cancer diagnosis [21]. Therefore, glycoproteins liberated from cancer nests are valuable for cancer diagnosis. Similarly, a sialic acid-targeted collection and detection approach may prove beneficial for the isolation of cancer cells and cancer-derived exosomes.

Sialic acid is a saccharide, and its concentrations can be measured by chemical or enzymatic reactions in the presence of sialic acid, colorimetric or fluorescent methods, or chromatography and mass spectrometry [22]. Selective binding to sialic acid for direct detection using various sugars is not as simple as antigen–antibody recognition. Boronic acid is a suitable candidate as a sensing probe for sialic acid detection because of its high specificity. Previous studies have shown that phenylboronic acid binds to diols in saccharides under basic conditions and to sialic acid under acidic conditions [23]. Because of this, boronic acids can selectively bind to sialic acid on glycoproteins to measure sialic acid concentrations on surfaces such as cancer cell membranes [24,25,26]. However, phenylboronic acid and sialic acid have weak binding constants (*K*_a_) of approximately 40 M^−1^ [23]. Consequently, in a previous study, the detection level for sialic acid using a boronic acid-functionalized metal organic framework was in the millimolar range [27]. Therefore, improvements in binding strength are required to effectively capture and collect cancer-derived targets.

Recently, heterocyclic boronic acids with high affinity, such as 5-boronopicolinic acid (5-BPA), have been explored as alternatives to phenylboronic acid. The *K*_a_ between 5-BPA and sialic acid is >1 × 10^3^ M^−1^ at pH 5.0, and this high affinity is maintained even when the carboxyl group is in an amide bond [28]. Practically, 5-BPA can be immobilized on sensing surfaces to facilitate effective sialic acid detection and collection. A strong binding affinity can be achieved through multisite binding, which functions similarly to a hook-and-loop fastener. In previous research, we found that a gold surface functionalized with 5-BPA could effectively detect sialic-acid-enriched glycoproteins in the nanomolar range [29]. Advantageously, the affinity of this boronic acid-based sialic acid binding system could be controlled using pH changes. The captured glycoproteins could be easily dissociated by changing the buffer pH from acidic to basic [29]. Therefore, 5-BPA-functionalized surfaces allow for the preparation of a collection system that can readily bind and release cancer-derived substances with changes in the pH.

**Figure 1:**
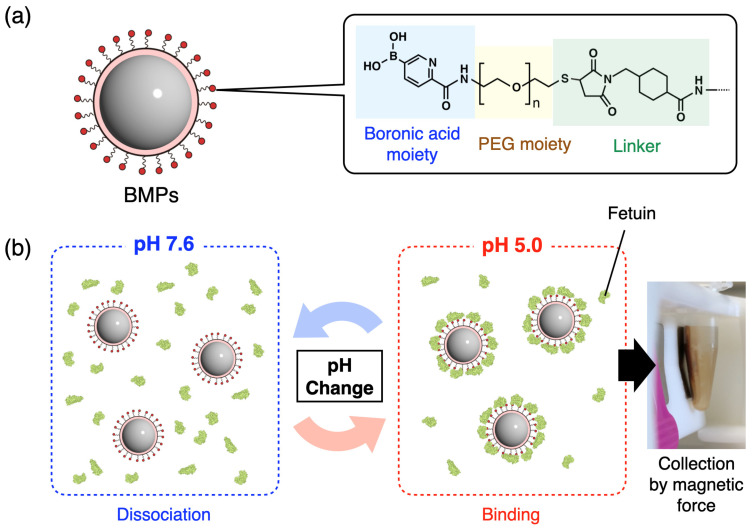
Outline of this work. (**a**) Design of the 5-BPA-modified magnetic particles (BMPs). 5-BPA was combined with polyethylene glycol (PEG) and then immobilized on magnetic particles. (**b**) Specific binding of fetuin as a model sialoglycoprotein on BMPs. Boronic acid moieties bind selectively to sialic acids such as *N*-acetylneuraminic acid (Neu5Ac) under acidic conditions. The sialoglycoprotein captured on BMPs can be easily dissociated by changing the pH to neutral or alkaline. When a complex of BMPs and fetuin forms, the fetuin can be purified and concentrated by collecting it using a magnet.

In this report, 5-BPA–modified magnetic particles (BMPs) were developed to demonstrate the functionality of a 5-BPA layer on surfaces. Functionalized small particles have a higher net surface area and are more likely to collide with the target than flat plates such as sensor surfaces, which shortens the reaction time and improves the final yield. It is possible to increase the reaction rate between the target species and the micro-sized particles by applying stirring or vibration. In practice, we achieved an efficient and rapid capture of sialic acid. Because of the magnetism of the microparticles, the target could be separated, purified, and concentrated from biological samples by modifying the surface of the microparticles with a ligand corresponding to the target. There have been numerous reports on the use of magnetic particles for medical applications because they provide magnetic guidance and can be used for monitoring and thermotherapy [30,31,32,33,34]. To evaluate the characteristics and performance of BMPs, fetuin was used in this study as a highly sialylated glycoprotein. To assess the practicality and usability of this method, we comprehensively evaluated the characteristics and performance of BMPs, including the pH responsiveness, capture efficiency, repeatability, and concentrating ability (Figure 1). Magnetic particles are prone to agglomeration. Previous reports have shown that modification with polyethylene glycol (PEG) inhibits agglomeration [35,36]. Therefore, PEG-modified magnetic particles are widely used for bioapplications [37,38,39]. The BMPs in this study were modified with PEG (Figure 1a).

## 2. Materials and Methods

### 2.1. Materials and Equipment

5-BPA was purchased from Combi-Blocks Inc. (San Diego, CA, USA). Heterobifunctional PEG (3.4 k) was obtained from Biopharma PEG Scientific Inc. (Watertown, MA, USA). Succinimidyl-4-(*N*-maleimidomethyl)cyclohexane-1-carboxylate (SMCC) was purchased from ProteoChem (Hurricane, UT). Trimethylamine, *N*,*N*-dimethylformamide (DMF), tris(2-carboxyethyl)phosphine) (TCEP) hydrochloride, 4-(4,6-dimethoxy-1,3,5-triazin-2-yl)-4-methylmorpholinium chloride n-hydrate, and 30% albumin solution from bovine serum were obtained from FUJIFILM Wako Pure Chemical Corporation (Osaka, Japan). Sera-Mag SpeedBead Blocked Amino Particles were purchased from Global Life Sciences Solutions Operations UK Ltd. (Cytiva, Buckinghamshire, UK). Spectra/Por 3 dialysis membrane standard RC tubing (molecular weight cutoff: 3.5 kDa; Repligen Corporation, Waltham, MA, USA) was used for the dialysis process. ^1^H-NMR spectroscopy was performed using an Avance400 (Bruker Corporation, Billerica, MA, USA). A PureProteome Magnetic Stand 8 well (Merck KGaA, Darmstadt, Germany) was used to collect the magnetic beads. An AdvanceBio Total Sialic Acid Quantitation Kit (Agilent Technologies, Inc., Santa Clara, CA, USA) and Varioskan LUX multimode microplate reader VLBL00D1 (Thermo Fisher Scientific, Inc., Waltham, MA, USA) were used to measure the quantity of released fetuin. Ultrapure water was prepared using a Milli-Q Reference system (Merck KGaA).

### 2.2. Synthesis of 5-BPA–Terminated PEG

**Figure 2:**
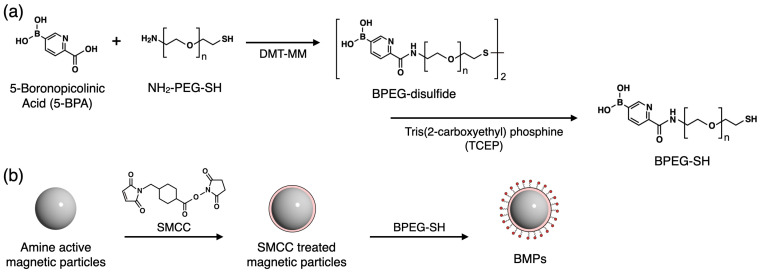
(**a**) Synthesis of 5-BPA–terminated PEG (BPEG-SH). (**b**) Preparation of BMPs.

5-BPA–terminated PEG (BPEG-SH) was synthesized as shown in Figure 2a. First, 0.2 mmol of 5-BPA (33.6 mg) was dispersed in 12.5 mL of DMF and the mixture was sonicated to achieve good dispersion. Triethylamine (175 μL) was mixed with the solution to facilitate condensation reactions, and then 0.025 mmol of heterobifunctional PEG (84 mg) was dissolved in the solution with sonication. 4-(4,6-Dimethoxy-1,3,5-triazin-2-yl)-4-methylmorpholinium chloride n-hydrate (18.2 mg, 0.055 mmol) was added to the solution for the condensation reaction, and the mixture was stirred for 4 h to prepare BPEG-disulfide. The BPEG-disulfide was dialyzed against 1 L of 100 mM NaOH overnight to exchange the solvent and remove the byproducts and unreacted 5-BPA (5-BPA is soluble under basic conditions). This dialysis was repeated three times, and then the product was dialyzed against 1 L of ultrapure water. The obtained product was reduced under vacuum and then lyophilized to completely remove the water. The yield was 76.5%. Because thiols readily oxidize to form disulfides, a reduction of BPEG-disulfide was performed using TCEP immediately before modification of the magnetic bead surface.

### 2.3. Preparation of the BMPs

The BMPs were prepared as shown in Figure 2b. Magnetic particles (MPs) with amino groups (5 × 10^11^ particle/mL, 1 mL) were trapped using a magnetic stand and then washed with ultrapure water to remove azide, which was used as a preservative. SMCC (5 mg) was dissolved in 100 μL of DMF and then mixed with the MPs for 1 h to prepare SMCC-MPs. The SMCC-MPs were then trapped using the magnetic stand and washed with ultrapure water to remove excess SMCC. Concurrently, 9.2 mg of 5-BPEG-disulfide was dissolved in ultrapure water and mixed with 100 μL of 200 mM TCEP-HCl to prepare BPEG-SH by reductive cleavage of the disulfide. The BPEG-SH solution and SMCC-MPs were then mixed for 1 h to prepare the BMPs. The BMPs were stored at 4 °C until required for further use.

### 2.4. Fetuin Collection Using the BMPs

The BMPs (4, 16 and 64 μL, 5 × 10^11^ particle/mL) and 1 mL of 1 mg/mL fetuin in an acidic solution (10 mM acetate buffer, pH 5.0) were placed in 1.5-mL microtubes, mixed, and incubated for 1 min. The BMPs were trapped using the magnetic stand. Unbound fetuin was removed, and the BMPs were washed twice with 1 mL of 10 mM acetate buffer. Fetuin-bound BMPs were redispersed in 50 μL of 10 mM phosphate buffer (pH 7.6) to dissociate fetuin from the BMPs. The BMPs were separated using the magnetic stand, and the supernatant containing the dissociated fetuin was collected. The amount of dissociated fetuin was measured using the AdvanceBio Total Sialic Acid Quantitation Kit. The same experiments were performed with a fetuin and BSA mixed solution (1 mg/mL for each component) for an inhibition test. BSA solution (1 mg/mL) was tested as a nonspecific sample. As a control experiment, 1 mg/mL of fetuin in a neutral solution (10 mM phosphate buffer, pH 7.6) was tested.

### 2.5. Repeatability of Binding and Dissociation between Fetuin and the BMPs

This procedure was similar to that described in Section 2.4. BMPs (64 μL, 5 × 10^11^ particle/mL) were mixed with 1 mL of 1 mg/mL fetuin solution (10 mmol/L acetate buffer, pH 5.0). The BMPs were trapped using the magnetic stand. After removing unbound fetuin, the BMPs were washed with 10 mmol/L acetate buffer. The BMPs were then redispersed in 50 μL of 10 mM phosphate buffer (pH 7.6) to dissociate the captured fetuin. After separation of the BMPs and fetuin, the remaining BMPs were mixed with 1 mL of 1 mg/mL fetuin solution (10 mM acetate buffer, pH 5.0) again. This process was repeated five times, and the amount of dissociated fetuin in each step was measured using the AdvanceBio Total Sialic Acid Quantitation Kit.

### 2.6. Condensation of Fetuin Using BMPs

The following solutions were prepared: fetuin solution (10 μg/mL) in 10 mmol/L acetate buffer (pH 5.0); fetuin solution (10 μg/mL) containing 1 mg/mL BSA in 10 mmol/L acetate buffer (pH 5.0); and fetuin solution (1 μg/mL) in 10 mmol/L acetate buffer (pH 5.0). Aliquots of these solutions (10 mL for the 10 μg/mL solutions and 100 mL for the 1 μg/mL solution) were separately mixed with 64 μL of BMPs (5 × 10^11^ particles/mL), and then the BMPs were collected using the magnetic stand. The collected BMPs were redispersed in 50 μL of 10 mmol/L phosphate buffer (pH 7.6) to dissociate the captured fetuin. After separation of the BMPs and fetuin, the amount of dissociated fetuin was measured using the AdvanceBio Total Sialic Acid Quantitation Kit.

## 3. Results and Discussion

### 3.1. Preparation of Boronic Acid-Terminated PEG

The design of the BMPs is shown in Figure 1a. An important factor to consider when preparing BMPs is the dispersion stability. Because 5-BPA has low solubility in water, the immobilization of 5-BPA on the MPs surface will prevent their stable dispersion. Reduced selectivity because of nonspecific adsorption should also be considered. PEG is a well-known polymer that maintains the dispersion stability of particles and prevents nonspecific interactions, such as reactions with sensing surfaces, because steric hydrophilic chains prevent the approach of impurities through the excluded volume effect. In this study, a PEG layer was added between 5-BPA and the MPs to improve the dispersion stability and prevent nonspecific interactions. To combine 5-BPA and PEG, amine-terminated PEG was used in a condensation reaction. The thiol group at the other end of the PEG chain was used for immobilization on the MPs. Because thiol groups are readily oxidized to disulfide groups, the reducing agent TCEP was used to reduce BPEG-disulfide to BPEG-SH (Figure 2).

**Figure 3:**
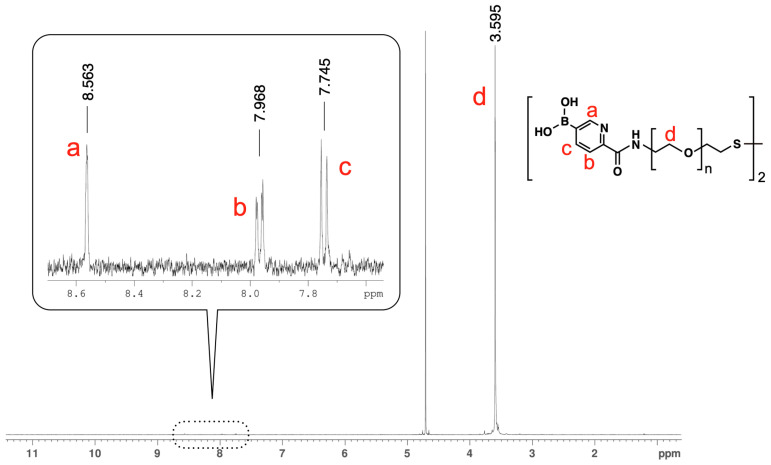
^1^H-NMR (NaOD/D_2_O, 400 MHz) spectrum of BPEG-disulfide. The a, b, c, d shown in the peaks correspond to the a, b, c, d protons indicated in the structural formula.

The rate of the condensation reaction between 5-BPA and amine-terminated PEG was evaluated by ^1^H-NMR after the removal of unreacted 5-BPA. Large peaks were observed for PEG (δ 3.595) and the solvent (water). Additionally, one singlet (peak a, δ 8.563, 1H) and two doublets (peak b, δ 7.968, 1H; and peak c δ 7.745, 1H) were observed for the 5-BPA moiety (Figure 3). These peaks were shifted compared with those for 5-BPA before the reaction (Figure S1). These shifts were likely caused by the condensation reaction. When unreacted 5-BPA was not completely removed, peaks for both reacted and unreacted 5-BPA were observed (Figure S2). However, for the sample in Figure 3, there was no unreacted 5-BPA. The ratio of the integrals of the 5-BPA and PEG peaks (peaks c and d in Figure 3) was 1:348.29. From this, the 5-BPA incorporation rate was estimated to be 94%.

### 3.2. Preparation of BMPs and Fetuin Capture Efficiency

Amine-activated MPs were reacted with SMCC, which is a conventional crosslinker. After the removal of unreacted SMCC, BPEG-SH was immobilized on MPs via a thiol-maleimide reaction (Figure 2). To confirm the fetuin capture efficiency of the BMPs, fetuin from fetal bovine serum was used as a highly sialylated model glycoprotein. After mixing the fetuin solution and BMPs in acetate buffer (pH 5.0), the BMPs were collected using a magnet and then washed with the buffer (Figure 1b). The fetuin captured on the BMPs was released in phosphate buffer (pH 7.6), and the liberated fetuin concentration was measured using the AdvanceBio Total Sialic Acid Quantitation Kit. To select appropriate pH values for binding and dissociation, we looked at data from previous reports rather than performing our own optimization experiments. For dissociation, the binding strength between boronic acid and sialic acid is reportedly minimized at pH 7 [28]. Therefore, we set the pH to 7.6 for the dissociation process. For binding, binding constants have been reported at pH values as low as pH 5 but not for lower pH values; however, the published curves suggest that the binding constant may increase with further lowering of the pH [28]. However, if the pH is too low, the original properties of the protein will be lost because it can denature. Therefore, we set the pH to 5 for binding.

**Figure 4:**
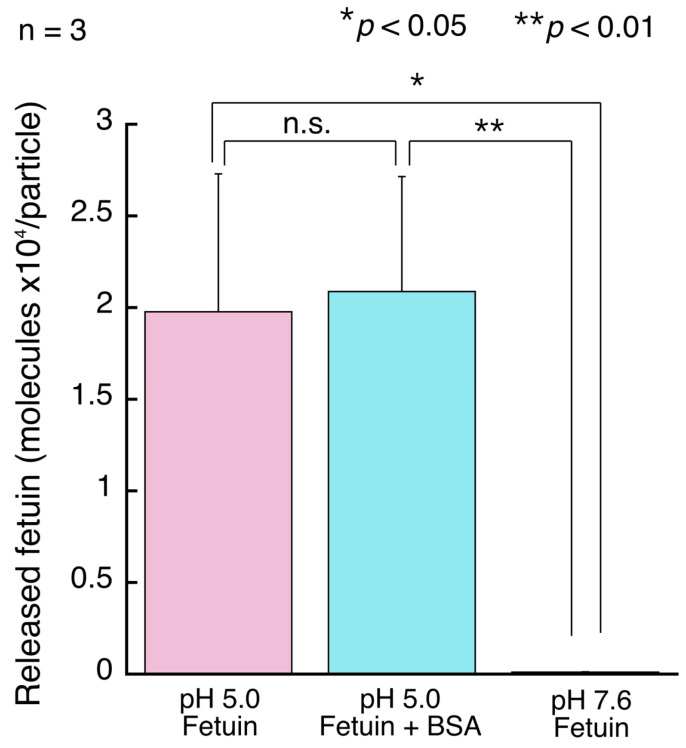
Estimation of the amount of released fetuin per BMP particle. The amount of fetuin released was measured, and then the number of molecules of fetuin released per BMP particle was calculated from the amount of BMP used. The plot shows the means of three independent tests ± standard deviations. * *p* < 0.05 and ** *p* < 0.01 for Student’s *t*-test. n.s. is an abbreviation for not significant.

The estimated average number of fetuin molecules bound per BMP particle was approximately 2.0 × 10^4^ molecules (Figure 4). Additionally, the number of fetuin molecules bound per BMP particle was almost the same, despite BSA being present as an impurity. By contrast, almost no fetuin was observed for the mixture of fetuin solution and BMPs in phosphate buffer (pH 7.6). This result shows that capture is caused by interactions between pH-responsive boronic acid and sialic acid, as previously reported [28,29]. We confirmed that BSA did not affect the results obtained with the AdvanceBio Total Sialic Acid Quantitation Kit (Figure S3). The diameter of the BMPs was approximately 1 μm (surface area: 3.14 × 10^6^ nm^2^); therefore, we estimated that one molecule of fetuin was bound per 150 nm^2^ on the surface of the BMP particles. This value is reasonable because the molecular weight of fetuin is 48.4 kDa.

### 3.3. Repeat Evaluation of Fetuin Capture and Release from BMPs

**Figure 5:**
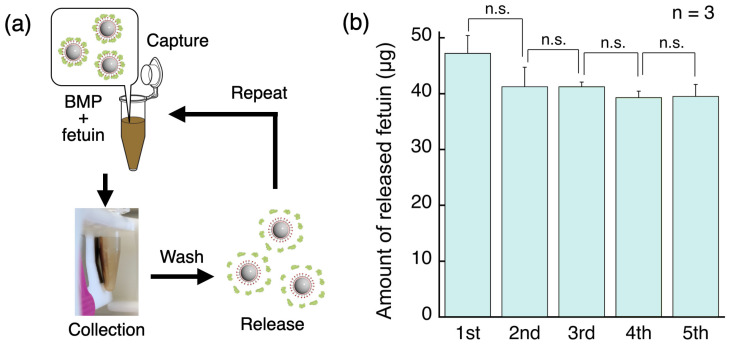
The repeatability of fetuin release from the BMPs. (**a**) The fetuin capture–release cycles. (**b**) Stability of the fetuin capture–release with the BMPs. The capture–release cycles were repeated five times. The plot shows the means of three independent tests ± standard deviations. n.s. is an abbreviation for not significant.

The sialic acid protein can be easily attached to and detached from the BMP surface by changing the pH, which means that it is easy to handle compared with antibodies. If this attachment and detachment can be repeated, the cost of collecting sialic acid protein will be dramatically reduced compared with conventional methods. Therefore, after dissociating fetuin, the collected BMPs were placed into the fetuin solution again. The ability of the BMPs to repeatedly capture fetuin was evaluated by carrying out repeated capture–release cycles (Figure 5a). There was a decrease in the amount of fetuin released (and therefore the amount of BMPs) between the first and the second cycles, but the changes after this were not significant. The BMPs could be used repeatedly, and their amount was maintained at 80% of the initial amount in the fifth cycle (Figure 5b). The decrease in the amount of BMPs that occurred between the first and second cycles may have been caused by some of the prepared BMPs lacking the required properties to withstand repeated manipulation with the magnet. Therefore, it is possible that they were lost between the first and the second cycles. By contrast, no significant changes were observed after the second cycle, which showed that the BMPs were highly reusable for capturing fetuin.

### 3.4. Effect of the Fetuin Concentration

One of the advantages of this system is the concentration of fetuin, which could potentially enhance the sensitivity for its detection. In early-stage cancers, detection is difficult because the levels of blood-circulating cancer cells or free cancer-derived proteins are extremely low. Sialic acid protein capture using BMPs could improve the detection performance. For example, it has been reported that prognosis is poor when more than five circulating tumor cells (CTCs) are present per 7.5 mL of blood [40]. Therefore, for the early detection of cancer, very low numbers of CTCs—only one in a few milliliters of blood—must be captured and concentrated.

**Figure 6:**
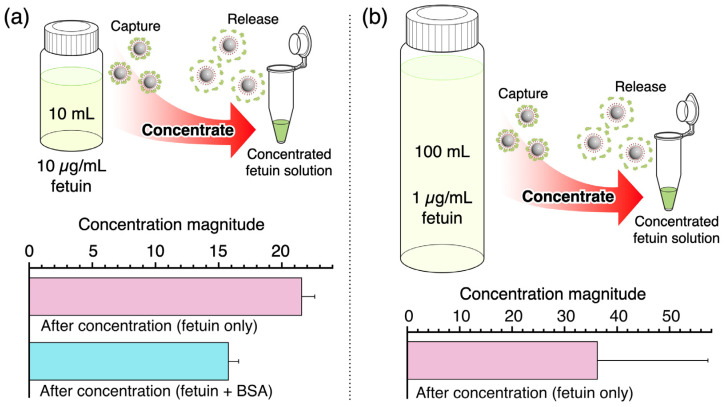
Fetuin concentration with BMPs. (**a**) A total of 10 mL of a 10 μg/mL fetuin solution was prepared and then concentrated using BMPs. To evaluate the concentration performance for an impure sample, a mixed solution (10 μg/mL fetuin and 1 mg/mL BSA) was used. (**b**) A 1 μg/mL fetuin solution was prepared, and 100 mL of this solution was concentrated using BMPs. The results presented are the mean of three independent tests ± standard deviation.

The concentration of a dilute solution of fetuin using the BMPs was demonstrated. Fetuin solution (10 mL of 10 μg/mL or 100 mL of 1 μg/mL) was mixed with BMPs. The fetuin on the BMP surface was then dissociated in 50 μL of phosphate buffer (pH 7.6). For the 10 μg/mL fetuin solution, the obtained fetuin concentration was >210 μg/mL, which was 21 times the prepared concentration (Figure 6a). Notably, the fetuin concentration efficacy was maintained at 16-fold even when the mass ratio of BSA–fetuin was 100:1, which indicated that the BMPs were highly selective. For the 1 μg/mL fetuin solution, the average fetuin concentration was approximately 36 μg/mL, which was 36 times the prepared fetuin concentration (Figure 6b). Although 10 mL of the 10 μg/mL fetuin solution and 100 mL of the 1 μg/mL fetuin solution contained the same total amount of fetuin, the concentration of collected fetuin was lower for the more dilute solution (36 μg/mL vs. 210 μg/mL).

For the 10 mL of 10 μg/mL fetuin solution (100 μg of fetuin), approximately 10.5 μg of fetuin was recovered by the BMPs. According to the repeatability of the fetuin release capacity of BMPs, approximately 40 μg of fetuin can be carried by the BMPs. Therefore, the recovery efficiency was approximately 25%. Consequently, the amount of fetuin bound to the BMPs decreased in dilute solutions. This observation could be explained by the binding–dissociation equilibrium. Equilibrium is reached at the point where the amount of dissociated fetuin and BMPs is higher in a low-concentration environment than in a high-concentration environment. Therefore, the amount of collected fetuin depends on the concentration of fetuin and BMPs. Another factor is the collection efficiency of the BMPs using the magnet. The prepared BMPs have both boronic acid moieties and PEG chains, which form a PEG layer on the BMP surface. This PEG layer prevents undesired nonspecific binding to BMPs and increases the dispersion stability. In a low-concentration solution, this characteristic is an obstacle to collecting BMPs using a magnet. The increase in the number of BMPs that were not collected by the magnet may have led to a decrease in the fetuin collection efficiency. This could be addressed by improving the experimental technique. In this experiment, manual collection was carried out using microtubes and a magnetic stand, and it was difficult to collect BMPs in the areas of the microtubes furthest from the magnet. The development of a collection system that uses narrow flow paths and allows for efficient interaction with the magnet could improve the efficiency of BMP recovery and increase the concentration effect. Additionally, BMPs can be used repeatedly; therefore, the collection rate could be improved by carrying out the concentration operation over multiple cycles. Another option might be to increase the amount of BMPs used in the experiment. Even though there is potential for further improvements, our results show that BMPs can concentrate a model sialoglycoprotein, which enables highly sensitive detection even in the low-concentration range.

Finally, we considered the potential uses of BMPs. An advantage of BMP-based concentration and purification technologies is that they can be combined with existing CTC and exosome extraction technologies. For example, when CTCs are separated from blood by centrifugation, this only removes red blood cells and plasma but not platelets and white blood cells. By contrast, BMPs selectively bind to CTCs overexpressing sialic acid and can be collected with a magnet. Therefore, BMPs could be used to efficiently collect and detect even very low numbers of CTCs. With exosomes, purification is performed by density gradient centrifugation. Any impurities contained within the fraction and exosomes that are not of cancer origin are also collected, which means that further purification is required. BMPs can selectively extract exosomes with sialic acid overexpression from samples with a high concentration of impurities. BMPs are expected to contribute to early diagnosis, for example, by selectively concentrating sialic acid-overexpressing cancer cells and cancer-derived biomolecules and increasing the concentrations of targets to detectable levels. Experiments conducted using fetuin as a model allowed us to demonstrate the basic mechanism of binding and dissociation for targeting sialic acid. However, the method was not applied to real samples. Real samples, such as cells and exosomes, are large and will bind over a larger area than fetuin. Consequently, the binding strengths between real samples and BMPs will differ from that between fetuin and BMPs. Therefore, the current results cannot be extrapolated to estimate a limit of detection for clinical samples. Previous reports have demonstrated that the boronic acid interface functions against sialic acids on the surface of cancer cells [25,26,41]. BMPs should be evaluated using real samples, such as metastatic cancer cells, in the future.

## 4. Conclusions

We developed a sialoglycoprotein collection method using BMPs. Because boronic acids bind to sialic acids under acidic conditions, the BMPs showed selective binding to fetuin, a glycoprotein with several sialic acid groups, at pH 5. Fetuin trapped on the BMP surface was dissociated by changing the pH to 7.6 and was quantified with a sialic acid kit. Furthermore, the binding and dissociation of boronic and sialic acids to/from the BMP surface was repeated by changing the pH, and fetuin was enriched by collecting the BMPs with a magnet. Metastatic cancer cells and exosomes derived from metastatic cancers overexpress sialic acids on their surfaces, which means that these cells and exosomes could be selectively concentrated and purified using BMPs. The proposed procedure, which is effective for condensing trace amounts of biomarkers in the blood so that they are detectable, could contribute to the early detection of cancer.

## Data Availability

The data presented in this study are available on request from the corresponding author (accurately indicate status).

## Supplementary Materials

The following supporting information can be downloaded at https://www.mdpi.com/article/10.3390/s24041080/s1, Figure S1: ^1^H-NMR spectrum of 5-boronopicolinic acid (5-BPA); Figure S2: ^1^H-NMR spectrum of BPEG-disulfide when the purification process was incomplete; Figure S3: Supplemental result of Figure 4.
