# Supplementary material for: Simple Binding and Dissociation of a Sialoglycoprotein Using Boronic Acid-Modified Functional Interfaces on Microparticles
Source: Sensors (Basel). 2024 Feb 7;24(4):1080. doi: 10.3390/s24041080 (PMC10891811; doi:10.3390/s24041080)
Supplement: Supplementary file 1 [file sensors-24-01080-s001.zip › sensors-2817205-supplementary.pdf]

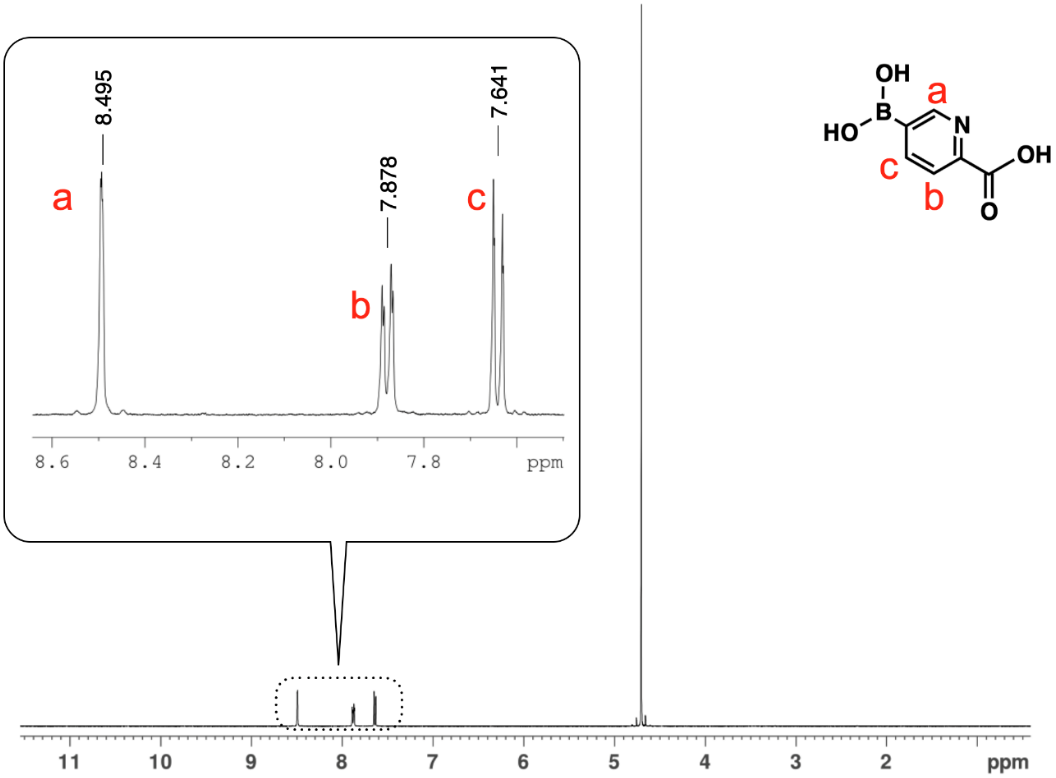

Figure S1. ^{1}H-NMR (NaOD/D_2O, 400 MHz) spectrum of 5-boronopicolinic acid (5-BPA). δ 8.495 (s, 1H), 7.878 (d, 1H), 7.631 (d, 1H) were observed. The a, b, c shown in the peaks correspond to the a, b, c protons indicated in the structural formula.

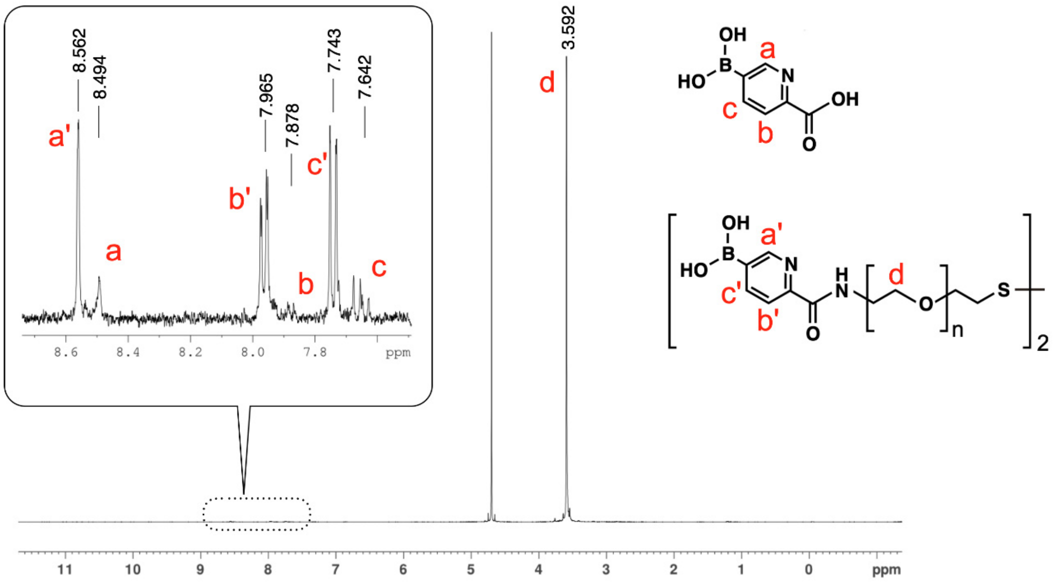

Figure S2. ^{1}H-NMR (NaOD/D_2O, 400 MHz) spectrum of BPEG-disulfide when the purification process was incomplete. Not only the peaks of BPEG-disulfide (δ 8.562 (s, 1H), 7.965 (d, 1H), 7.743 (d, 1H), and large peak derived from PEG at 3.592), but the peaks of unreacted 5-BPA (δ 8.494 (s, 1H), 7.878 (d, 1H), 7.642 (d, 1H)) were also observed. The a, a', b, b', c, c′, d shown in the peaks correspond to the a, a', b, b', c, c′ d protons indicated in the structural formula. The result clearly shows that the peaks of BPA were shifted from (a, b, c) to (a', b', c') after the reaction.

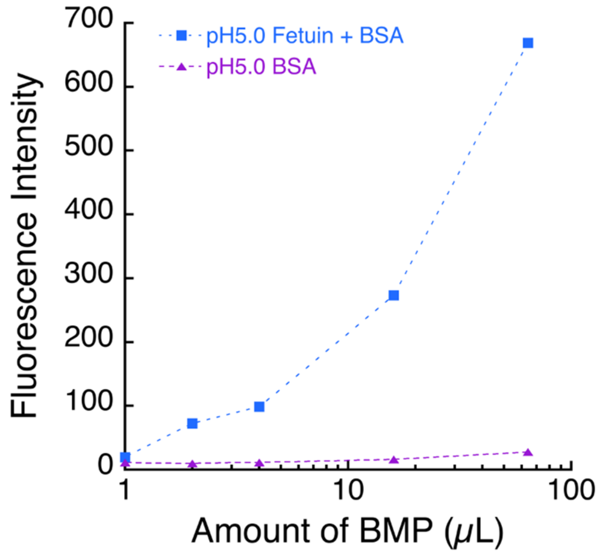

Figure S3. Supplemental result of Figure 4. Almost no fluorescence was observed in the experiment in which BSA solution was mixed with BMPs. Therefore, it was confirmed that BSA did not affect the results of the sialic acid quantification.
